# Bullatine A suppresses glioma cell growth by targeting SIRT6

**DOI:** 10.1016/j.heliyon.2024.e41440

**Published:** 2024-12-24

**Authors:** Zhi Wang, Yushuai Zhu, Can Luo, Fan Zhang, Jiannong Zhao, Chuanyi Fu

**Affiliations:** a Department of Cerebrovascular Disease, Hainan General Hospital, Hainan Affiliated Hospital of Hainan Medical University, Haikou, Hainan 570311, PR China; b Department of Neurosurgery, Hainan General Hospital, Hainan Affiliated Hospital of Hainan Medical University, Haikou, Hainan 570311, PR China

**Keywords:** Bullatine a, Glioma, Cell proliferation, Apoptosis, SIRT6

## Abstract

Gliomas are the most common primary tumors of the nervous system, which is generally treated using adjuvant chemotherapy following surgical resection. However, patient survival time is still short, and there is currently no successful treatment for highly malignant gliomas. Bullatine A (BLA) is a diterpenoid alkaloid of the genus Aconitum which antirheumatic and anti-inflammatory pharmacological properties. The effects of BLA on gliomas have not yet been elucidated. In this study, we investigated the effects of BLA on human brain malignant glioblastoma cells. Our results showed that BLA inhibited the proliferation of U87MG and U251 cells in a dose-dependent manner and decreased their survival rate. BLA dose-dependently induced apoptosis in U87MG cells, upregulated the expression of cleaved caspase-9, cleaved caspase-3 pro-apoptotic protein, and Bax protein, and downregulated the expression of Bcl-2 anti-apoptotic protein. Moreover, BLA dose-dependently induced U87MG and U251 cell cycle arrest in the G2/M phase, and downregulated the expression of p-ERK and Myc proteins. Further, BLA significantly inhibited the acetylation of histones H3K9 and H3K56, and upregulated the expression of the protein deacetylase SIRT6. Mechanistic studies revealed that the effect of BLA on inducing apoptosis and inhibiting the proliferation of glioma cells was blocked by SIRT6 knockout. In summary, our study indicated that BLA is a potential therapeutic agent for glioma that targets SIRT6 to inhibit glioma cell proliferation and induce apoptosis.

## Highlights

• Bullatine A induces apoptosis in human brain malignant glioblastoma cells.
• Bullatine A inhibits glioma cells proliferation by inducing G2/M cell cycle arrest.
• Bullatine A induces apoptosis and inhibits growth by promoting SIRT6 expression.

## Graphical abstract

**Figure:**
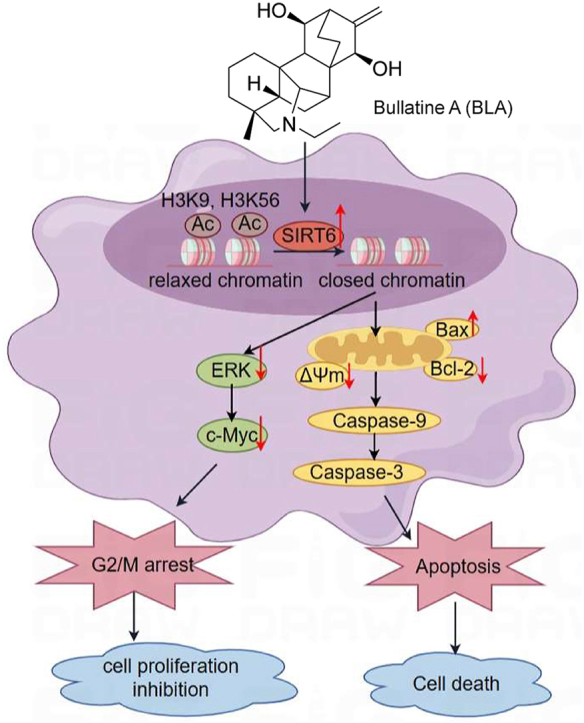

## 1 Introduction

Gliomas are common primary tumors in the brain which usually originate from glial or precursor cells; these tumors account for approximately 30 % of all brain and central nervous system tumors, and 80 % of all malignant brain tumors [1]. Gliomas are typically characterized by uncontrolled proliferation, invasion, and angiogenesis [2], and can progress to astrocytomas, oligodendrogliomas, ventricular meningiomas, or oligodendrocytes [3]. In 2016, the WHO Health Organization classified gliomas according to their malignancy grade and histological type into astrocytoma of grades I, II, III or IV, the latter also being known as glioblastoma (GBM). GBM represents the deadliest brain cancer, with a median survival of fewer than 2 years [4]. Currently, commonly used GBM treatments include surgery, radiotherapy, chemotherapy, and combination therapy, but it is characterized by tumor recurrence and chemoresistance. A significant reason for this is due to the high cell heterogeneity of GBM, including Proneural/Neural, Classical, and Mesenchymal subtypes [[5], [6], [7], [8]]. Additionally, the intricate complex tumor microenvironment (TME) contributes to these challenges, with interactions occurring between tumor cells and neurons, astrocytes, oligodendrocytes, macrophages, microglia, and other immune populations within the central nervous systems (CNS) [[9], [10], [11], [12]]. To counteract this issue, some small-molecule inhibitors have been studied, such as ZnPP, Vemurafenib, VP18/58 targeting heme oxygenase-1, Haloperidol, BD1047 targeting sigma receptors. Despite these research advancements, prognosis in glioma patients remains poor [13,14,15,16], searching for new drugs to treat gliomas is necessary to provide new ideas and strategies for treating the disease.

Epigenetic regulation is essential to sustain life. Histone acetylation is one of the most relevant modifications in an organism's physiological functions and pathological phenotypes. Class III histone deacetylases (HDACs), also known as sirtuins (SIRT), are a highly conserved class of proteins [17]. In mammals, there are seven forms of sirtuins, classified into classes I to IV, based on their structural differences [18]: specifically, SIRT1, SIRT2, and SIRT3 belong to class I; SIRT4 to class II; SIRT5 to class III; and SIRT6 and SIRT7 to class IV. The SIRT protein family plays a vital role in the development of gliomas. Studies shows SIRT1 in glioma has been a tumor-promoting role. It has been found that SIRT1 may promote glioma cell proliferation and inhibit apoptosis via the PTEN/PI3K/AKT signaling pathway [16]. Further, recent studies have shown that SIRT6 is generally considered to have a suppressive role in gliomas. It inhibits glioma cell growth by inducing apoptosis, inhibiting oxidative stress, and suppressing JAK2/STAT3 signaling pathway activation [19]. Another study showed that SIRT6 plays an important role in maintaining genomic stability and preventing the accumulation of DNA damage, thereby inhibiting tumor progression [20,21]. Considering the differences, targeting SIRT6 may be considered a novel strategy for treating gliomas.

Bullatine A (BLA), a diterpenoid alkaloid of the genus Aconitum, exerts both antirheumatic and anti-inflammatory effects [22]. In regards to the latter effect, BLA inhibits part of the inflammatory response through the ROS/JNK/NF-κB signaling pathway [23]. Further, BLA has been shown to inhibit ATP-induced apoptosis in microglia by antagonizing the P2X7 receptor [22]. However, reports on the antitumor effects of BLA are lacking.

In the present study, we found that BLA inhibits proliferation and induces apoptosis of glioma cells. In addition, BLA treatment triggered the upregulation of apoptosis-related proteins, and downregulation of anti-apoptotic proteins in glioma cells. Furthermore, we found that BLA could deacetylate two histones, H3K9Ac and H3K56Ac, by upregulating the expression of SIRT6, thus inhibiting cancer progression. Overall, our results indicate that BLA can inhibit the proliferation and promote the apoptosis of glioma cells, indicating it as a potential drug for the treatment of glioma.

## 2 Methodology

### 2.1 Reagents

Bullatine A was obtained from Shanghai TopScience Co., Ltd (Shanghai, China); DMEM medium and Trypsin-EDTA were obtained from Gibco (New York, USA); Penicillin-Streptomycin solution, Cell counting kit-8, and 4 % paraformaldehyde fix solution were obtained from Biosharp (Hefei, Anhui, China); fetal bovine serum was obtained from Cegrogen Biotech (Stadtallendorf, Germany); dimethyl sulfoxide (DMSO) was obtained from Beijing Solarbio Science & Technology Co., Ltd (Beijing, China); and BeyoECL star, crystal violet staining solution, HRP-conjugated secondary antibody (anti-Mouse, Cat. A0216 and anti-Rabbit, Cat. A0208) were obtained from Beyotime Biotechnology (Shanghai, China). Antibodies against cleaved caspase-9 (#9508), cleaved caspase-3 (#9661), Bcl-2 (#3498), Bax (#2772), GAPDH (#2118), phospho-ERK (#4370), T-ERK (#4695), Myc (#13987), H3K9Ac (#9649), Histone protein 3 (#9715), SIRT6 (#2590), LaminB (#13435) were purchased from Cell Signaling Technology (Danvers, Massachusetts, USA). H3K56Ac antibody (#39282) was purchased from WUHAN SANYING Co., Ltd. (Wuhan, Hubei, China). The Annexin V-FITC/PI apoptosis kit (AP101C), Cell Cycle Staining Kit (CCS012) were purchased from Multisciences Biotech Co., Ltd. (Hangzhou, Zhejiang, China).

### 2.2 Cell culture

Human glioblastoma cells U87MG were purchased from ATCC (HTB-14, Manassas, Virginia, USA), and U251 were purchased from Nanjing Cobioer Biosciences Co., Ltd. (CBP60300, Nanjing, China). Cells were cultured in complete medium comprising DMEM supplemented with 10 % fetal bovine serum and 1 % penicillin-streptomycin solution. The cells were cultured in an incubator at 37 °C with 5 % CO2.

### 2.3 CCK-8 experiment

Cells under good growth conditions underwent digestion and centrifugation. Subsequently, cells were resuspended in DMEM complete medium, at a cell concentration adjusted to 1 × 10^5^ cells/ml, after which 100 μl of cell suspension was added to the wells of 96-well plates. After 24 h, the supernatant of each well was aspirated, 100 μl of 10 % CCK-8 solution in complete medium was added to each well, and the plate was placed in an incubator at 37 °C for 2 h. The absorbance at 450 nm was subsequently measured using a microplate reader.

### 2.4 Cell colony formation assay

Human glioblastoma cells with good growth status in the logarithmic growth stage were digested with trypsin-EDTA and centrifuged, after which the supernatant was discarded, and cells were resuspended in complete DMEM. A total of 600 cells/well were counted and plated in a 6-well plate with 0, 50, 200, or 800 nM BLA solution added to each well. The 6-well plates were incubated at 37 °C in a 5 % CO2 incubator for 7 days. Subsequently, cell colonies were stained with 0.005 % crystal violet and analyzed under a microscope.

### 2.5 Cell cycle assay

Human glioblastoma cells were diluted to a density of 5 × 10^5^ cells/well and inoculated into a 6-well plate. After treatment, the cells were harvested and fixed in anhydrous ethanol (pre-chilled in a −20 °C refrigerator) overnight at −20 °C. The fixed samples were then stained with a DNA staining solution for 30 min at room temperature in the dark. Stained cells were analyzed using a flow cytometer (Thermo Fisher Scientific, Shanghai, China).

### 2.6 Annexin V-FITC/PI apoptosis detection

U87MG human glioblastoma cells were resuspended in pre-chilled 1 × binding buffer at a concentration of 1 × 10^6^ cells/ml. Subsequently, 1 × 10^5^ cells were stained with 5 μl Annexin V-FITC and 10 μl PI, and incubated for 5 min at room temperature protected from light. Apoptosis was determined by flow cytometry (Thermo Fisher Scientific).

### 2.7 Mitochondrial membrane potential assay

U87MG human glioblastoma cells were stained with MitoPT JC-1 (ImmunoChemistry Technologies, Bloomington, MN). Briefly, after treatment with BLA for 24 h, cells were harvested by centrifugation and incubated with JC-1 staining solution for 30 min at 37 °C in the dark after which cells were rinsed twice with JC-1 buffer and then resuspended in PBS. Then, stained cells were analyzed using flow cytometry (Thermo Fisher Scientific, Shanghai). A plot of red fluorescence from living cells with intact mitochondrial membrane potential and green fluorescence from cells with loss of mitochondrial membrane potential was recorded.

### 2.8 Western blot analysis

U87MG human glioblastoma cells were inoculated at a density of 5 × 10^5^ cells/well in a 6-well plate and treated with BLA at concentrations of 0, 5, 15, and 45 μM for 24 h. The cells were subsequently lysed by adding appropriate amounts of RIPA buffer and PMSF (Phenylmethylsulfonyl fluoride) (RIPA: PMSF = 100:1). The concentration of each protein group was determined using bicinchoninic acid protein quantification, followed by sodium (sulfate-polyacrylamide gel) protein electrophoresis, membrane transfer, and incubation with primary and secondary antibodies (1:1000 dilution for 1 h). Finally, chromatograms were developed using the BeyoECL Star kit, exposed to an instrument Tanon-5200 for color development, and photographed for analysis/storage.

### 2.9 shRNA transfection

Human SIRT6 shRNA was purchased from Shanghai GeneChem Co., Ltd. The shRNA control (scramble) sequence was 5′-TTCTCCGAACGTGTCACGT-3’. U87MG cells in 6-well plates (50–70 % confluent) were transfected with the shRNA plasmid using Lipofectamine 3000, according to the manufacturer's instructions.

### 2.10 Real-time qPCR analysis

Real-time qPCR analysis was performed to compare the levels of gene expression using a real-time polymerase chain reaction with the SYBR Premix Ex *Taq*II kit (TaKaRa), followed by detection using the ABI Prism 7000 Sequence Detection System (Applied Biosystems). Total RNA was extracted from cells using the TRIzol reagent (Invitrogen). The samples (100 ng) were then amplified using specific primers. The amplification primers for SIRT6 were purchased from GeneChem (Shanghai, China). The primers for GAPDH were as follows: sense, TGGGCTACACTGAGCACCAG, antisense, GGGTGTCGCTGTTGAAGTCA.

### 2.11 Statistical analysis

All experiments were performed in triplicate. Graphing and analysis were performed using Graph Pad Prism 8.0 software. Data are expressed as the mean ± SEM, and statistical differences were compared using either Student's *t*-test or one -way ANOVA. Statistical significance was set at P < 0.05.

## 3 Results

### 3.1 Bullatine A suppresses glioma cell proliferation and colony formation

**Fig. 1:**
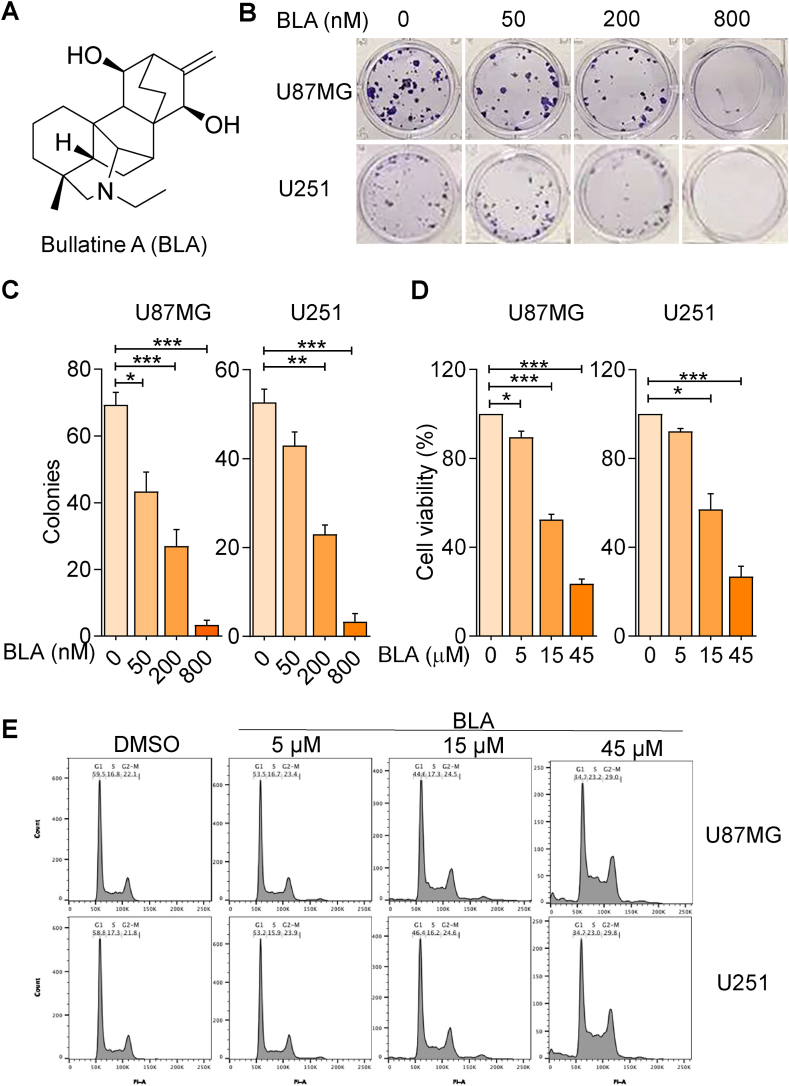
**Bullatine A suppresses glioma cell proliferation and colony formation.** (**A**) The chemical structure and formula of BLA. (**B**) Representative images of colonies on colony formation assay. Cells were treated with BLA at concentrations of 0, 50, 200, and 800 nM. (**C**) Quantification of the colony numbers of U87MG and U251 cells in **B**. (**D**) Cell viability was analyzed by CCK-8 assay after 24 h of BLA treatment. (**E**) U87MG and U251 cells were treated with 5, 15, and 45 μM BLA for 24 h, after which cell cycle distribution was determined by flow cytometry. The results are expressed as the mean ± SEM of three independent experiments (n = 3, ∗P < 0.05, ∗∗P < 0.01 and ∗∗∗P < 0.001 compared with control).

To investigate the role of Bullatine A (BLA) in glioma proliferation, we performed colony formation and CCK-8 assays to measure the colony formation capability and cell viability of human glioma U87MG and U251 cells. Glioma cells were treated with Bullatine A (BLA) at concentrations ranging from 50 to 800 nM. As presented in Fig. 1A and B, Bullatine A treatment significantly decreased the ability of U87MG and U251 cells to grow clonally in a dose-dependent manner. Assessment of number of clonal cell clusters revealed that cell proliferation was almost completely inhibited at a drug concentration of 800 nM (Fig. 1B and C). Further, CCK-8 assay revealed a significant decrease in cell viability when U87MG and U251 cells were treated with BLA at the concentration of 15 and 45 μM (Fig. 1D). Subsequently, we examined the effects of BLA on the cell cycle of glioma cells, finding that the percentage of U87MG and U251 cells in the G1 phase decreased significantly following treatment with BLA compared to DMSO-treated control cells. Conversely, the proportion of cells in the G2/M phase was remarkably increased compared to DMSO-treated controls following exposure to 15–45 μM BLA (Fig. 1E). Taken together, these results suggest that BLA inhibits glioma cell proliferation by blocking cell cycle progression.

### 3.2 Bullatine A induces glioma cell apoptosis

**Fig. 2:**
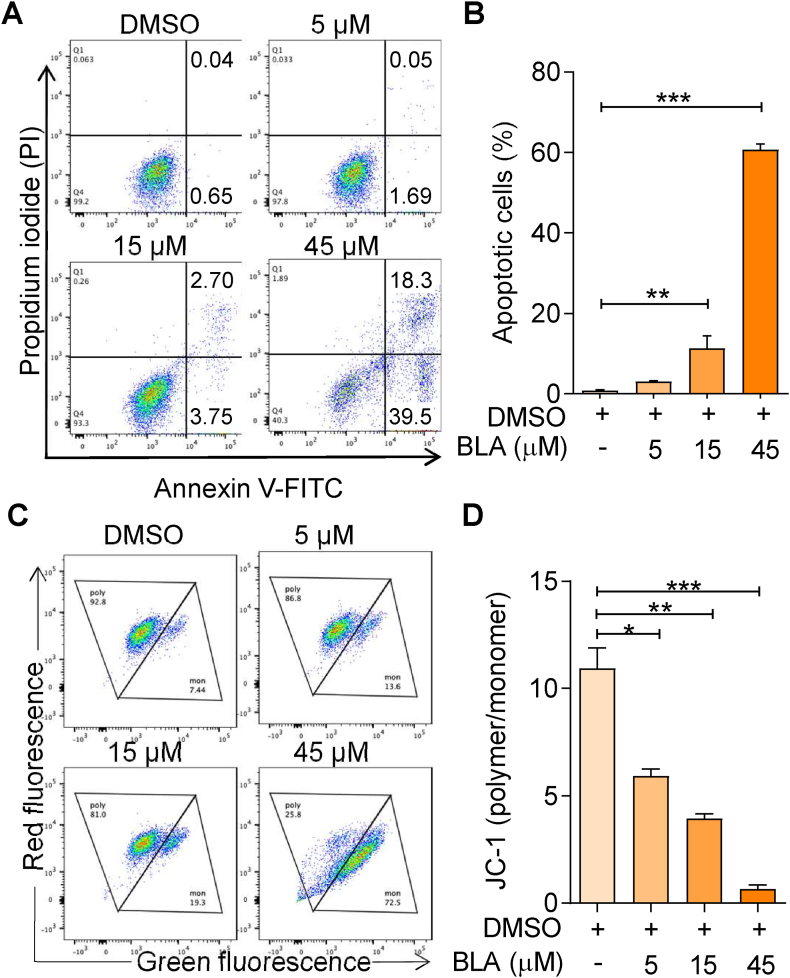
**Bullatine A induces glioma cell apoptosis.** (**A**) Analysis of apoptosis in U87MG cells after treatment with BLA (5, 15, and 45 μM) for 24 h, followed by Annexin V-FITC/PI double staining with flow cytometry detection. (**B**) Quantification of the percentage of living cells. (**C**) U87MG cells were incubated with different concentrations of BLA (5, 15, and 45 μM) for 24 h, after which the change of mitochondrial membrane potential (MMP) was determined using JC-1 staining and flow cytometry analysis. (**D**) The ratio of polymer to monomer intensity were analyzed to assess the quantitative change of MMP. The results are expressed as the mean ± SEM of three independent experiments (n = 3, ∗P < 0.05, ∗∗P < 0.01 and ∗∗∗P < 0.001 compared with control).

To investigate whether BLA regulated glioma apoptosis, we performed FACS to detect apoptotic cells. U87MG cells were treated with BLA at concentrations of 0, 5, 15, and 45 μM for 24 h, and subsequently stained with Annexin V-FITC/PI. As presented in Fig. 2A, treatment of U87MG cells with BLA increased the proportion of PI^−^/Annexin V^+^ early apoptotic cells and PI^+^/Annexin V^+^ late apoptotic cells. Statistical analysis of early (quadrant IV) and late (quadrant I) apoptotic cells revealed that the apoptotic rate increased significantly in a dose-dependent manner (Fig. 2B). A decrease in mitochondrial membrane potential is deemed a hallmark event for early apoptosis; therefore, we stained the cells with JC-1, a mitochondria-specific dye, to detect the changes in the mitochondrial membrane potential (MMP) of U87MG cells after treatment with BLA at 0, 5, 15, and 45 μM. As shown in Fig. 2C, BLA treatment significantly decreased the amount of JC-1 polymer. Statistical comparison of MMP loss was performed using JC-1 polymer quantity and the JC-1 polymer/monomer ratio. The results revealed that U87MG cells underwent a significant loss of MMP affected by BLA after 24 h of BLA treatment in a dose-dependent manner (Fig. 2D). Taken together, these results suggest that BLA-induced apoptosis in U87MG cells may be related to mitochondrial dysfunction.

### 3.3 Bullatine A regulates Bax signaling in human glioma cells

**Fig. 3:**
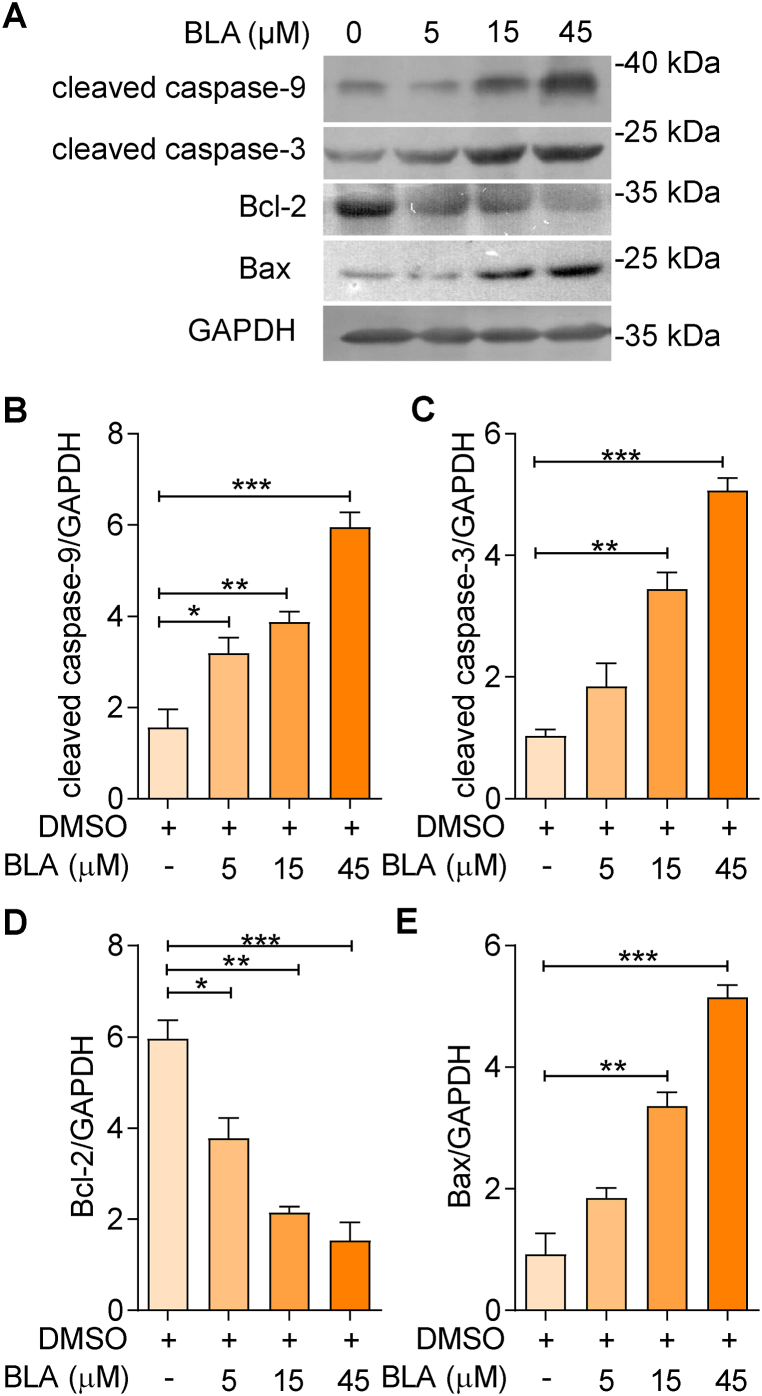
**Bullatine A regulates Bax signaling in human glioma cells.** (**A**) U87MG cells were pretreated with BLA at 0, 5, 15, and 45 μM for 24 h. The protein levels of cleaved caspase-9, cleaved caspase-3, Bcl-2, and Bax were subsequent assessed by western blot. (**B**–**E**): Quantitative analysis of apoptosis-related protein expression. The results are expressed as the mean ± SEM of three independent experiments. ∗P < 0.05, ∗∗P < 0.01, ∗∗∗P < 0.001 compared with the 0 μM group. Source data are provided in supplementary file.

To further investigate the mechanism of apoptosis, we examined the expression levels of pro- and anti-apoptotic proteins [24] following BLA treatment in U87MG cells. Western blot results showed that the expression of cleaved caspase-9, cleaved caspase-3, and the pro-apoptotic protein Bax were all significantly increased in BLA-treated U87MG cells compared to the control group. In contrast, expression of the anti-apoptotic protein Bcl-2 decreased in U87MG cells after 24 h of treatment with BLA (Fig. 3A). Statistical analyses revealed a dose-dependent increase in cleaved caspase-9, cleaved caspase-3, and Bax protein levels as well as a dose-dependent decreased in Bcl-2 protein level induced by BLA (Fig. 3B–E). Taken together, these data suggest that the mitochondrial apoptotic pathway plays an important role in BLA-induced apoptosis.

### 3.4 Bullatine A regulates ERK1/2 signaling in human glioma cells

**Fig. 4:**
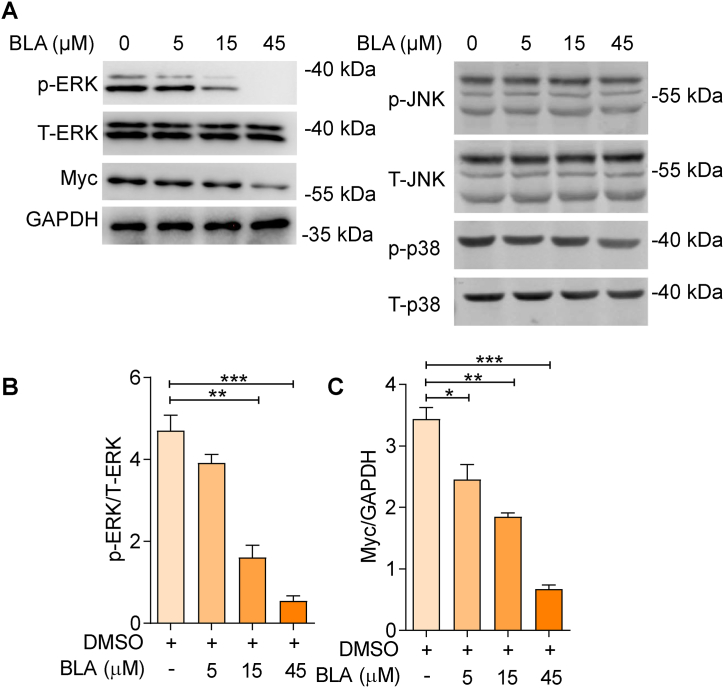
**Bullatine A regulates ERK1/2 signaling in human glioma cells.** (**A**) U87MG cells were pretreated with BLA at 0, 5, 15, and 45 μM for 24 h. The protein levels of p-ERK and Myc were measured by western blot. (**B**–**C**): Quantitative analysis of apoptosis-related protein expression. The results are expressed as the mean ± SEM of three independent experiments. ∗P < 0.05, ∗∗P < 0.01, ∗∗∗P < 0.001 compared with the 0 μM group. Source data are provided in supplementary file.

ERK is a member of the MAPK signaling pathway, the downregulation of which leads to decreased cell viability. Myc is a vital tumor regulator whose downregulation leads to cell cycle arrest and promotes apoptosis. To further explore the regulatory effects of BLA on gliomas, the levels of ERK phosphorylation and Myc total protein were determined in BLA-treated U87MG cells. The data illustrated that BLA significantly downregulated the p-ERK and Myc levels in BLA-treated U87MG cells, with little effect on JNK and p38 pathways (Fig. 4A). Statistical analyses revealed a dose-dependent alteration in p-ERK and Myc levels induced by BLA (Fig. 4B and C). Taken together, our data indicated the inhibitory potential of BLA against ERK/Myc signaling.

### 3.5 Bullatine A regulates the acetylation levels of H3K9 and H3K56, and the protein level of SIRT6

**Fig. 5:**
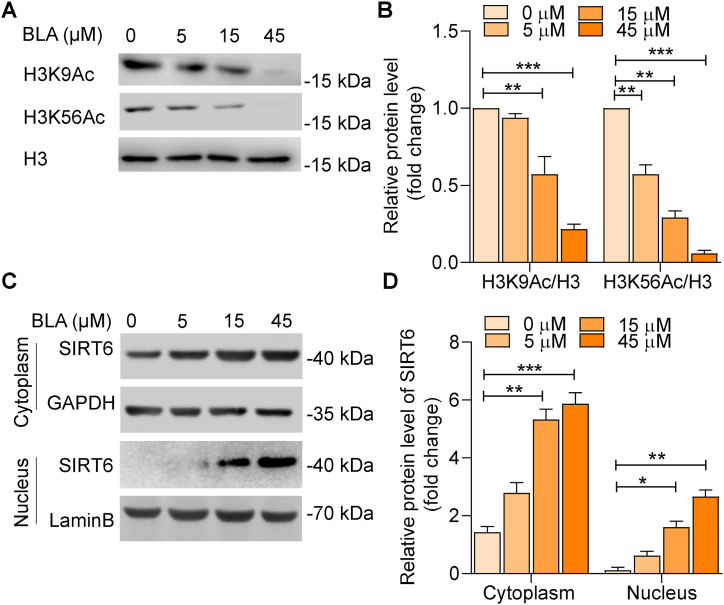
**Bullatine A regulates the acetylation level of H3K9 and H3K56, and the protein level of SIRT-6.** (**A**) U87MG cells were pretreated with BLA at 0, 5, 15, and 45 μM for 24 h. The acetylation levels of H3K9 and H3K56 were subsequently assessed by western blot. (**B**) Quantification of the blots in **A**. (**C**) The protein level of SIRT 6 in cytoplasm and nucleus expression was measured by western blot. (**D**) Quantitative analysis of SIRT6 protein expression. The results are expressed as the mean ± SEM of three independent experiments. ∗P < 0.05, ∗∗P < 0.01, ∗∗∗P < 0.001 compared with the 0 μM group. Source data are provided in supplementary file.

Histone modification by acetylation is an important epigenetic feature and one of the modifications most closely linked to an organism's physiological function and pathological characterization. Based on recent studies showing that cellular levels of histone modifications are linked to glioma prognosis, we investigated the epigenetic changes involved in the inhibition of cell proliferation and apoptosis. Our results revealed that BLA significantly inhibited the expression of two acetylated histones, H3K9Ac and H3K56Ac, in a dose-dependent manner (Fig. 5A and B). As SIRT6, a member of the histone deacetylase family, is known to play a major role in the deacetylation of H3K9 and H3K56, we subsequently examined whether BLA regulates the expression of SIRT6. Our results revealed that BLA significantly increased both the cytoplasm and nucleus expression of SIRT6 protein in a dose-dependent manner (Fig. 5C and D). Taken together, these findings indicate that BLA may inhibit tumor development by targeting SIRT6 to deacetylate histones H3K9Ac and H3K56Ac.

### 3.6 SIRT6 deficiency blocks the effects of BLA on glioma cell proliferation and apoptosis

**Fig. 6:**
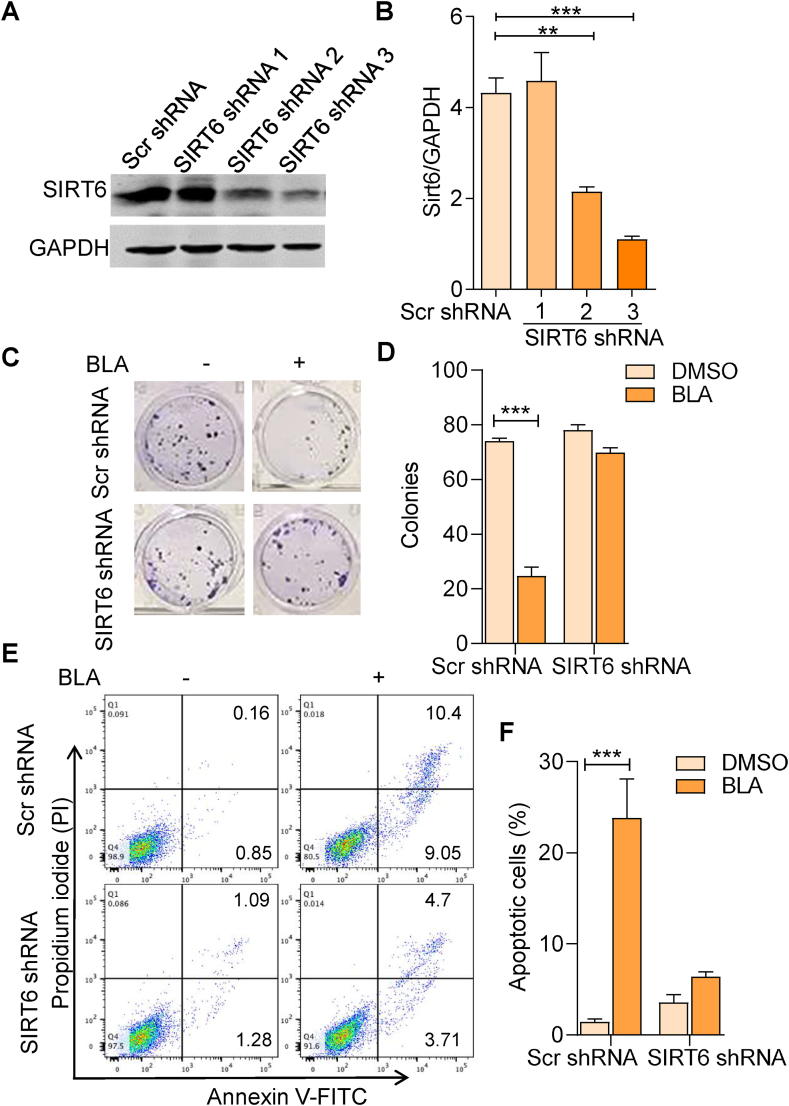
**SIRT6 deficiency blocks the effects of BLA on glioma cell proliferation and apoptosis.** (**A**) Immunoblot detection of SIRT6 protein expression in U87MG cells transfected with different SIRT6 shRNAs. (**B**) Quantitative analysis of SIRT6 proteins. (**C**) U87MG cells were transfected with SIRT6 shRNA3 for 24 h. Subsequently, SIRT6 knockdown cells were treated with BLA (45 μM) for 24 h. Representative images of colonies by colony formation assay. (**D**) Quantification of the colony numbers of U87MG cells in **C**. (**E**) Apoptosis in SIRT6 deficient U87MG cells and control cells following treatment with BLA for 24 h were detected by Annexin V-FITC/PI double staining. (**F**) Quantification of the percentage of living cells in **E**. The results are expressed as the mean ± SEM of three independent experiments. ∗∗∗P < 0.001 compared with the control group. Source data are provided in supplementary file.

To further investigate whether BLA induces apoptosis and inhibits the proliferation of U87MG cells through SIRT6, we down-regulated the expression of SIRT6 in U87MG cells using SIRT6 specific shRNA. As presented in Fig. 6A, the protein level of SIRT6 was significantly inhibited in SIRT6 shRNA3-treated U87MG cells; therefore, we selected SIRT6 shRNA3 for further experiments. Colony formation and apoptosis in SIRT6 knockdown U87MG cells after pretreatment with BLA were assessed. As presented in Fig. 6C and D, proliferation of Scr shRNA U87MG cells was inhibited after BLA treatment. However, BLA did not affect the proliferation of SIRT6 knocked-down U87MG cells. In addition, we found that BLA had no effect on U87MG cell apoptosis in the absence of SIRT6 (Fig. 6E and F). Collectively, these results indicate that BLA induces the expression of SIRT6 to modulate the acetylation levels of H3K9 and H3K56, and subsequently regulates glioma cell proliferation and apoptosis.

## 4 Discussion

In this study, we discovered that BLA, a C20-diterpenoid alkaloid, significantly inhibited proliferation and induced apoptosis in glioma cells. In addition, BLA increased the expression of apoptosis-related proteins, including cleaved caspase-9, cleaved caspase-3, and Bax, and further inhibited the expression of the anti-apoptotic protein Bcl-2 and the proliferation-related proteins Myc and p-ERK. Mechanistic studies revealed that BLA regulates glioma cell proliferation and apoptosis by increasing the expression of SIRT6 and then inhibiting H3K9 and H3K56 acetylation.

BLA is a diterpenoid alkaloid of the genus Aconitum that exerts anti-rheumatic, anti-inflammatory, and anti-injury sensing effects [22]. Although the solubility of Bullatine A is generally poor in water but many previous reports showed that BLA can better in organic solvents, entering cells through the lipid bilayer of the cell membrane and exert it effects. For example, in vitro studies have shown that high concentrations of ATP acting on microglia (BV-2) activate P2X receptors and pro-inflammatory cytokines, ultimately triggering inflammation and neuronal death [25]. BLA is a potent P2X7 antagonist that inhibits ATP-induced apoptosis and P2X receptor-mediated inflammatory responses [22]. Macrophages and microglia are innate immune cells in the body's defense system that play a crucial role in the inflammatory response by releasing pro-inflammatory cytokines [26]. Indeed, prior studies have shown that BLA can effectively inhibit the overexpression of inflammatory factors in macrophages and microglia by inhibiting the ROS/JNK/NF-κB signaling pathway, thus exerting anti-inflammatory effects [23]. BLA can also specifically stimulate spinal microglia to express and secrete prednisolone A, by interacting with κ-opioid receptors on the postsynaptic neurons of microglia to exert antinociceptive hyperalgesic responses [27]. In addition to its anti-inflammatory and analgesic effects, in our study we found that BLA also has antitumor properties. Our analyses revealed that BLA promoted apoptosis and inhibited glioma cell proliferation, while increasing the expression of apoptosis-related proteins, including cleaved caspase-9, cleaved caspase-3, and Bax, and inhibiting the expression of the anti-apoptotic protein Bcl-2 to suppress the proliferation of glioma cells and induce a G2/M cell cycle arrest. Thus, the present study shows that BLA may exert a powerful antitumor effect, mainly by improving glioma cell proliferation and apoptosis.

ERK is a member of the mitogen-activated protein kinase (MAPK) family, a crucial signaling pathway that regulates various cellular processes, including proliferation, differentiation, apoptosis, and stress responses. Phosphorylation-activated ERK1/2 translocates from the cytoplasm to the nucleus, subsequently mediating the transcriptional activation of ELK1, ATF1, CREB, c-Fos, and c-Myc. Myc, which functions as a transcriptional regulators of cell growth, cell cycle, differentiation, apoptosis, and angiogenesis, is one of the most widely studied oncogenes [28]. Dysregulation of Myc gene expression has been identified in up to 70 % of human cancers [29]. In the present study, we found that BLA downregulated the phosphorylation of ERK1/2 and Myc in U87MG cells. Thus, BLA treatment may be a potential new strategy for treating malignancies by targeting Myc.

Histone acetylation is a dynamic chromatin marker that plays an important role in gene regulation, DNA damage repair, and DNA replication. Through its pleiotropic activity, histone acetylation can directly or indirectly regulate cells, and the determine critical processes associated with tumorigenesis such as DNA damage repair, metabolic homeostasis, and apoptosis. Indeed, prior studies have shown that the inhibition of histone acetylase can suppress the growth of glioma cells and induce glioma cell to undergo apoptosis. For example, histone acetylation suppresses human glioma cell proliferation by enhancing the expression of p21 and gelsolin [30]. In the present study, we found that the acetylation of H3K9 and H3K56 was significantly inhibited by BLA treatment. Given that histone acetylation of H3K9 and H3K56 is generally regulated by the deacetylase SIRT6 [[21], [22], [23]], we postulated that deacetylation of H3K9 and H3K56 may be mediated by alterations in SIRT6 expression. In the present study, we found that BLA upregulated SIRT6 protein expression in U87MG cells. In subsequent experiments, we further demonstrated that the effect of BLA on glioma cell proliferation and apoptosis was greatly blocked by the downregulation of SIRT6 expression, indicating that SIRT6 is required for the antitumor effects of BLA on U87MG cells. Increasing evidence has further shown that SIRT6 has a two-sided effect on tumor progression [21]. For example, SIRT6 plays an oncogenic role in osteosarcoma [20] and papillary thyroid cancer [26]. However, it has also been reported that icariin can up-regulate the expression of SIRT6 protein and deacetylates H3K9 on the promoter of the NF-κB target gene to induce apoptosis and inhibit tumor cell metastasis via SIRT6/NF-κB in triple-negative breast cancer [20]. As such, SIRT6 activation or inhibition may be beneficial depending on other specific type of cancer. However, the effects of BLA on different types of cancers requires further investigation.

In summary, our study revealed that BLA abrogates human glioma cell proliferation and induces cell cycle arrest and apoptosis, in a manner potentially related to mitochondrial dysfunction. Furthermore, we found that the antitumor activity of BLA relied on the induction of SIRT6 expression and histone deacetylation. Overall, our study indicated that BLA may serve as a potential therapeutic drug for gliomas.

## CRediT authorship contribution statement

**Zhi Wang:** Writing – original draft, Formal analysis, Data curation. **Yushuai Zhu:** Software, Formal analysis, Data curation. **Can Luo:** Software, Formal analysis, Data curation. **Fan Zhang:** Methodology, Formal analysis. **Jiannong Zhao:** Methodology, Conceptualization. **Chuanyi Fu:** Project administration, Funding acquisition, Conceptualization.

## Data availability statement

The data presented in this study are available in the article.

## Declaration of competing interest

The authors declare that they have no known competing financial interests or personal relationships that could have appeared to influence the work reported in this paper.

## References

[1] Goodenberger M.L., Jenkins R.B. (2012). Genetics of adult glioma. Cancer Genet.

[2] Calderon M.A., Bousquet J., Canonica G.W., Cardell L.O., Fernandez de Rojas D.H., Kleine-Tebbe J., Demoly P. (2017). Guideline recommendations on the use of allergen immunotherapy in house dust mite allergy: time for a change?. J. Allergy Clin. Immunol..

[3] Ostrom Q.T., Gittleman H., Truitt G., Boscia A., Kruchko C., Barnholtz-Sloan J.S. (2018). CBTRUS statistical report: primary brain and other central nervous system tumors diagnosed in the United States in 2011-2015. Neuro Oncol..

[4] Weller M., Le Rhun E. (2020). How did lomustine become standard of care in recurrent glioblastoma?. Cancer Treat Rev..

[5] Verhaak R.G., Hoadley K.A., Purdom E., Wang V., Qi Y., Wilkerson M.D., Miller C.R., Ding L., Golub T., Mesirov J.P., Alexe G., Lawrence M., O'Kelly M., Tamayo P., Weir B.A., Gabriel S., Winckler W., Gupta S., Jakkula L., Feiler H.S., Hodgson J.G., James C.D., Sarkaria J.N., Brennan C., Kahn A., Spellman P.T., Wilson R.K., Speed T.P., Gray J.W., Meyerson M., Getz G., Perou C.M., Hayes D.N., Cancer N. (2010). Genome Atlas Research, Integrated genomic analysis identifies clinically relevant subtypes of glioblastoma characterized by abnormalities in PDGFRA, IDH1, EGFR, and NF1. Cancer Cell.

[6] D'Amico A.G., Maugeri G., Vanella L., Pittala V., Reglodi D., D'Agata V. (2021). Multimodal role of PACAP in glioblastoma. Brain Sci..

[7] Wang Q., Hu B., Hu X., Kim H., Squatrito M., Scarpace L., deCarvalho A.C., Lyu S., Li P., Li Y., Barthel F., Cho H.J., Lin Y.H., Satani N., Martinez-Ledesma E., Zheng S., Chang E., Sauve C.G., Olar A., Lan Z.D., Finocchiaro G., Phillips J.J., Berger M.S., Gabrusiewicz K.R., Wang G., Eskilsson E., Hu J., Mikkelsen T., DePinho R.A., Muller F., Heimberger A.B., Sulman E.P., Nam D.H., Verhaak R.G.W. (2017). Tumor evolution of glioma-intrinsic gene expression subtypes associates with immunological changes in the microenvironment. Cancer Cell.

[8] Xu C., Hou P., Li X., Xiao M., Zhang Z., Li Z., Xu J., Liu G., Tan Y., Fang C. (2024). Comprehensive understanding of glioblastoma molecular phenotypes: classification, characteristics, and transition. Cancer Biol Med.

[9] Virtuoso A., Giovannoni R., De Luca C., Gargano F., Cerasuolo M., Maggio N., Lavitrano M., Papa M. (2021). The glioblastoma microenvironment: morphology, metabolism, and molecular signature of glial dynamics to discover metabolic rewiring sequence. Int. J. Mol. Sci..

[10] Maugeri G., D'Amico A.G., Saccone S., Federico C., Rasa D.M., Caltabiano R., Broggi G., Giunta S., Musumeci G., D'Agata V. (2021). Effect of PACAP on hypoxia-induced angiogenesis and epithelial-mesenchymal transition in glioblastoma. Biomedicines.

[11] Di Ianni N., Maffezzini M., Eoli M., Pellegatta S. (2021). Revisiting the immunological aspects of temozolomide considering the genetic landscape and the immune microenvironment composition of glioblastoma. Front. Oncol..

[12] Virtuoso A., D'Amico G., Scalia F., De Luca C., Papa M., Maugeri G., D'Agata V., Caruso Bavisotto C., D'Amico A.G. (2024). The interplay between glioblastoma cells and tumor microenvironment: new perspectives for early diagnosis and targeted cancer therapy. Brain Sci..

[13] D'Amico A.G., Maugeri G., Vanella L., Consoli V., Sorrenti V., Bruno F., Federico C., Fallica A.N., Pittala V., D'Agata V. (2024). Novel acetamide-based HO-1 inhibitor counteracts glioblastoma progression by interfering with the hypoxic-angiogenic pathway. Int. J. Mol. Sci..

[14] Romeo G., Ciaffaglione V., Amata E., Dichiara M., Calabrese L., Vanella L., Sorrenti V., Grosso S., D'Amico A.G., D'Agata V., Intagliata S., Salerno L. (2021). Combination of heme oxygenase-1 inhibition and sigma receptor modulation for anticancer activity. Molecules.

[15] Fallica A.N., Sorrenti V., D'Amico A.G., Salerno L., Romeo G., Intagliata S., Consoli V., Floresta G., Rescifina A., D'Agata V., Vanella L., Pittala V. (2021). Discovery of novel acetamide-based heme oxygenase-1 inhibitors with potent in vitro antiproliferative activity. J. Med. Chem..

[16] Chen C., Chi H., Sun B.G., Sun L. (2013). The galectin-3-binding protein of Cynoglossus semilaevis is a secreted protein of the innate immune system that binds a wide range of bacteria and is involved in host phagocytosis. Dev. Comp. Immunol..

[17] Imai S., Armstrong C.M., Kaeberlein M., Guarente L. (2000). Transcriptional silencing and longevity protein Sir2 is an NAD-dependent histone deacetylase. Nature.

[18] Frye R.A. (2000). Phylogenetic classification of prokaryotic and eukaryotic Sir2-like proteins. Biochem. Biophys. Res. Commun..

[19] Deng Q., Wang W., Sun L., Wang Y., Liao J., Xu D., Liu Y., Ye R., Gooneratne R. (2017). A sensitive method for simultaneous quantitative determination of surfactin and iturin by LC-MS/MS. Anal. Bioanal. Chem..

[20] Chen Y.M., Zheng Y., Yu Y., Wang Y., Huang Q., Qian F., Sun L., Song Z.G., Chen Z., Feng J., An Y., Yang J., Su Z., Sun S., Dai F., Chen Q., Lu Q., Li P., Ling Y., Yang Z., Tang H., Shi L., Jin L., Holmes E.C., Ding C., Zhu T.Y., Zhang Y.Z. (2020). Blood molecular markers associated with COVID-19 immunopathology and multi-organ damage. EMBO J..

[21] Fiorentino F., Carafa V., Favale G., Altucci L., Mai A., Rotili D. (2021). The two-faced role of SIRT6 in cancer. Cancers.

[22] Li J., Ren W., Huang X.J., Zou D.J., Hu X. (2013). Bullatine A, a diterpenoid alkaloid of the genus Aconitum, could attenuate ATP-induced BV-2 microglia death/apoptosis via P2X receptor pathways. Brain Res. Bull..

[23] Li Y., Xu Y.J., Tan C.P., Liu Y. (2022). Sinapine improves LPS-induced oxidative stress in hepatocytes by down-regulating MCJ protein expression. Life Sci..

[24] Brown J.M., Attardi L.D. (2005). The role of apoptosis in cancer development and treatment response. Nat. Rev. Cancer.

[25] Florenzano F., Viscomi M.T., Amadio S., D'Ambrosi N., Volonte C., Molinari M. (2008). Do ATP and NO interact in the CNS?. Prog Neurobiol.

[26] Chen X., Xu Z., Zeng S., Wang X., Liu W., Qian L., Wei J., Yang X., Shen Q., Gong Z., Yan Y. (2019). SIRT5 downregulation is associated with poor prognosis in glioblastoma. Cancer Biomark.

[27] Huang Q., Mao X.F., Wu H.Y., Li T.F., Sun M.L., Liu H., Wang Y.X. (2016). Bullatine A stimulates spinal microglial dynorphin A expression to produce anti-hypersensitivity in a variety of rat pain models. J. Neuroinflammation.

[28] Beaulieu M.E., Castillo F., Soucek L. (2020). Structural and biophysical insights into the function of the intrinsically disordered Myc oncoprotein. Cells.

[29] Dang C.V. (2012). MYC on the path to cancer. Cell.

[30] Kamitani H., Taniura S., Watanabe K., Sakamoto M., Watanabe T., Eling T. (2002). Histone acetylation may suppress human glioma cell proliferation when p21 WAF/Cip1 and gelsolin are induced. Neuro Oncol..

