# Supplementary figures and images for: Bullatine A suppresses glioma cell growth by targeting SIRT6
Source: Heliyon. 2024 Dec 24;11(1):e41440. doi: 10.1016/j.heliyon.2024.e41440 (PMC11750491; doi:10.1016/j.heliyon.2024.e41440)

**Figure 3A**

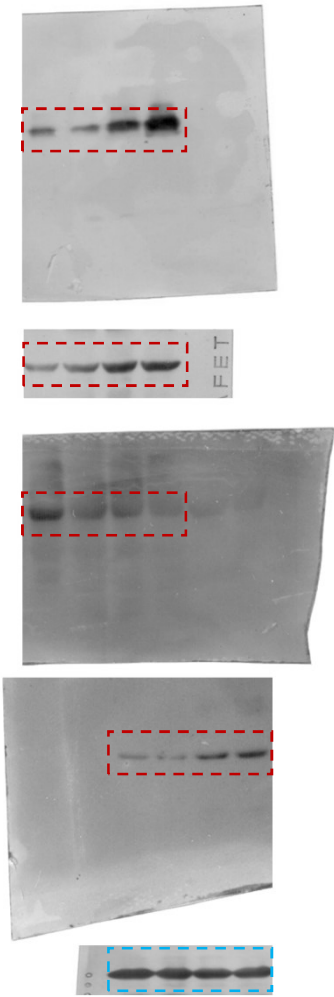

**Figure 4A**

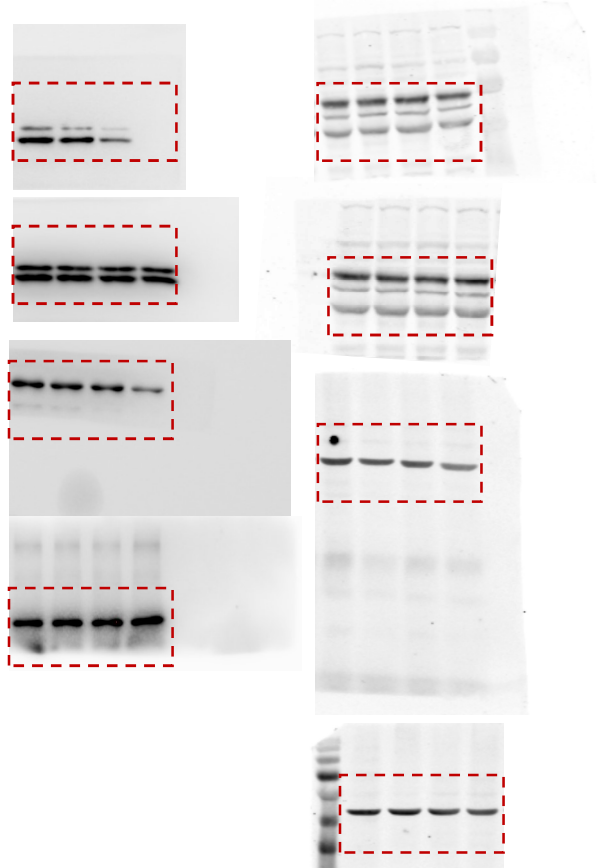

**Figure 5A**

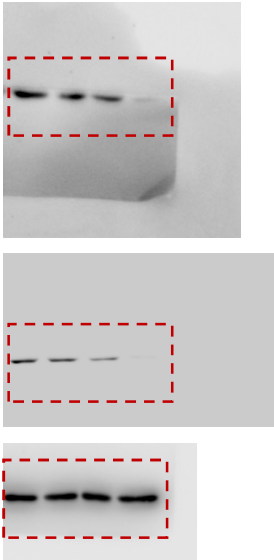

**Figure 5C**

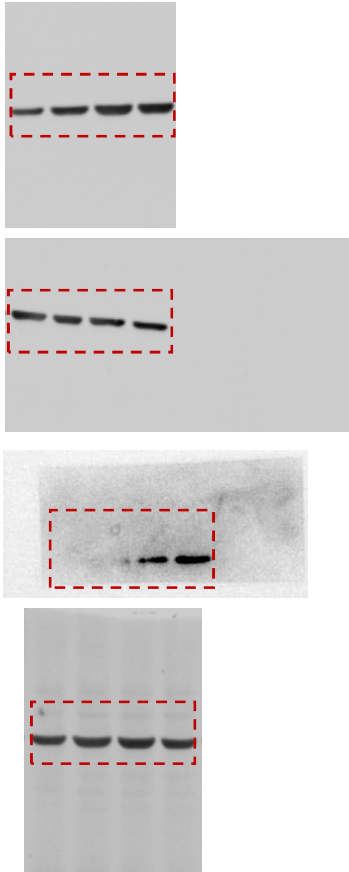

**Figure.6A**

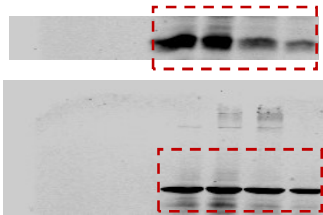

Supplement: Multimedia component 1 [file mmc1.pdf]
